# Pelvic floor dysfunction one year after first childbirth in relation to perineal tear severity

**DOI:** 10.1038/s41598-021-91799-8

**Published:** 2021-06-15

**Authors:** Malin Huber, Ellen Malers, Katarina Tunón

**Affiliations:** 1 Department of Clinical Sciences, Obstetrics and Gynecology, Umeå University, 901 87 Umeå, Sweden; 2 Department of Clinical Sciences, Obstetrics and Gynecology, Unit of Obstetrics and Gynecology-Östersund, Umeå University, Umeå, Sweden

**Keywords:** Sexual dysfunction, Urinary incontinence, Risk factors, Preventive medicine, Quality of life

## Abstract

The aims of this study were to evaluate pelvic floor dysfunction symptoms one year after delivery and investigate whether adverse functional outcomes after childbirth were related to the degree of perineal injury. A prospective cohort of 776 primiparas were included. Self-reported pelvic floor function data were obtained using a web-based questionnaire. Women with no/first-degree injuries, second-degree injuries, third-/fourth-degree injuries (obstetric anal sphincter injury, OASI) and cesarean section were compared. A total of 511 women (66%) responded. Second-degree tears were a risk factor for stress incontinence (aOR 2.6 (95% CI 1.3–5.1)). Cesarean section was protective against stress incontinence (aOR 0.2 (95% CI 0.1–0.9)). OASI was a risk factor for urge incontinence (aOR 4.8 (95% CI 1.6–15)), prolapse (aOR 7.7 (95% CI 2.1–29)) and pelvic pain (OR 3.3 (95% CI 1.1–10)). Dyspareunia was reported by 38% of women, 63% of women in the OASI group (aOR 3.1 (95% CI 1.1–9.0)). Women with OASI reported that the injury affected daily life (OR 18 (95% CI 5.1–59)). Pelvic floor dysfunction is common after childbirth, even in women with moderate injury. Women with OASI had significantly higher risks of symptoms of prolapse, urge urinary incontinence, pain, dyspareunia and impacts on daily life.

## Introduction

Approximately 80% of primiparas suffer from perineal laceration. An estimated 40–50% of lacerations involve the perineal muscles, and up to 7% of these women suffer from severe obstetric anal sphincter injury (OASI)^1,2^. The prevalence rates of minor (first-degree) and muscular (second-degree) lacerations are estimated according to clinical experience, and these data are not always recorded in patient charts or registers. OASI is the most common cause of anal incontinence among women. Grade two and OASI can lead to perineal pain and dyspareunia and increase the risk of concomitant pelvic floor disorders^3^. The association between previous birth-related pelvic floor muscle trauma and future signs of pelvic organ prolapse has been shown in long-term follow-up studies^4^. The odds of fecal or urinary incontinence four years after delivery are markedly higher among women who experience pelvic disorder symptoms in the first year after birth^5^. Perineal trauma has been shown to relate to the frequency and severity of postpartum dyspareunia^6^.

Research has primarily focused on the risk factors and complications of OASI, although vast areas in the field remain unexplored^7^. There is also a lack of knowledge about the extent of short-term complications following moderate spontaneous or iatrogenic injuries to the perineal muscles.

The aims of this study were to evaluate symptoms of prolapse, urinary and anal incontinence, and perineal pain as well as sexual function one year after birth and investigate whether adverse functional outcomes were related to the degree of perineal injury. We hypothesized that the prevalence of symptoms of postpartum pelvic floor dysfunction would be related to the degree of perineal trauma.

## Results

**Figure 1:**
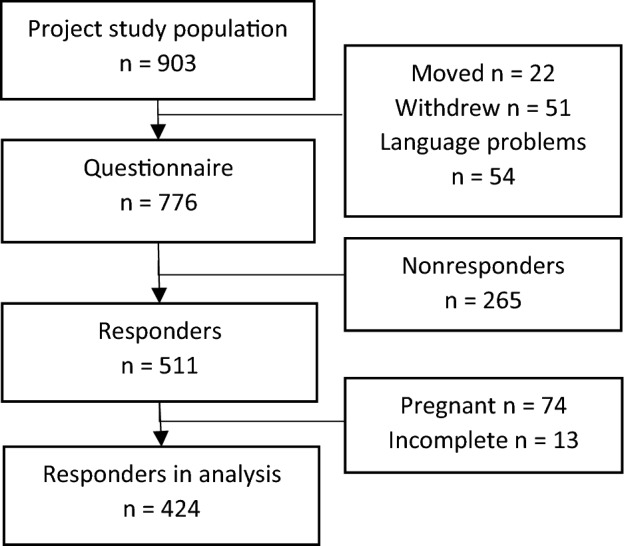
Flow chart of the study population.

A flowchart of participant retention in the study is presented in Fig. 1. The one-year follow-up questionnaire was distributed to 776 women, and 511 participants completed the questionnaire, accounting for a response rate of 66%. Of these participants, 74 (14.2%) were excluded from the analysis of symptoms due to de novo pregnancy, and 12 (2.3%) datasets were illegible/incomplete. Table 1 shows the descriptive statistics of the responders and nonresponders. There were no differences between the women who participated in the follow-up and the nonresponders regarding rates of perineal trauma. The respondents were older, and among them, the vacuum delivery rate was higher. Epidural use was more common among nonresponders than among responders (Table 1).

**Table 1:** Demographic and obstetric characteristics of the study population.

Characteristics	Responders n = 511	Nonresponders n = 265	*p* value
**Maternal factors**			
Maternal age (years), mean	30.3 (± 4.5)	28.6 (± 5.0)	< 0.001
Body mass index (kg/m^2^), mean	25.0 (± 4.6)	24.7 (± 4.2)	0.323
Smoking	2 (0.4)	4 (1.5)	na
**Fetal factors**			
Birthweight (g), mean	3459 (± 564)	3426 (± 528)	0.428
Head circumference (cm), mean	34.8 (± 2.3)	34.7 (± 1.5)	0.309
pH umbilical artery, mean	7.27 (± 0.08)	7.26 (± 0.08)	na
Presentation			
Occiput anterior	448 (87.7)	235 (88.7)	0.682
Occiput posterior	26 (5.1)	9 (3.4)	0.282
Breech, n	19 (3.7)	10 (3.8)	na
**Obstetric factors**			
Labor onset			
Spontaneous	390 (76.3)	197 (74.3)	0.542
Induction	95 (18.6)	56 (21.1)	0.396
Elective cesarean	26 (5.1)	12 (4.5)	0.732
**Labor outcomes**			
Length of active secondary state (min)	32	32	na
Oxytocin	325 (63.6)	181 (68.3)	0.217
Epidural	304 (59.5)	187 (70.6)	0.002
Vacuum extraction	61 (11.9)	15 (5.7)	0.005
Episiotomy	101 (19.8)	47 (17.7)	0.495
Cesarean section	69 (13.5)	43 (16.2)	0.306
**Perineal trauma**			
No/1st-degree injury	216 (42.3)	117 (44.2)	0.616
2nd-degree injury, n	201 (39.3)	97 (36.6)	0.458
3rd-/4th-degree injury	25 (5.0)	8 (3.1)	0.22

Data are presented as the mean ± standard deviation (SD) or n (%).

* na = not analyzed.

The demographic and obstetric characteristics of the responders in the three different perineal trauma groups and the cesarean section group are presented and compared in Table 2. There were no differences among the three injury groups regarding maternal age, body mass index, infant head circumference, gestational age at delivery, induction rate, labor augmentation or epidural use. The duration of the active secondary stage of labor was significantly longer in patients with second-degree injuries than in those with an intact perineum or first-degree injuries. There was a significant difference in infant birthweight between patients with no or minor perineal trauma and OASI, with the highest infant birthweight among women affected by OASI. Occiput posterior presentation was more common in the OASI group than in the other groups, but the difference was not significant after post hoc testing.

**Table 2:** Demographic and obstetric characteristics of analyzed responders in the three different groups of perineal trauma and cesarean section.

Characteristics	No injury/1st degree n = 176 (41.5)	2nd degree n = 175 (41.3)	OASIS n = 19 (4.5)	Cesarean section n = 54 (12.7)	*p* value
**Maternal factors**					
Maternal age (years)	30 (± 4.4)	30 (± 4.6)	31 (± 3.6)	32 (± 4.8)	0.072
Body mass index (kg/m^2^)	25 (± 4.1)	25 (± 4.4)	26 (± 4.2)	26 (± 5.3)	0.177
**Fetal factors**					
Birthweight (g)	3415 (± 503)	3513 (± 494)	3786(± 617)	3349(± 802)	0.009
Head circumf. (cm)	34.7 (± 2.1)	34.8 (± 2.7)	35.0 (± 1.5)	35.1 (± 2.2)	0.758
Ph Umbilical artery	7.27 (± 0.08))	7.27 (± 0.08)	7.26(± 0.07)	7.24 (± 0.07)	0.103
Presentation					
Occiput anterior	170 (97.1)	165 (93.8)	16 (84.2)	21 (38.9)	< 0.001
Occiput posterior	4 (2.3)	11 (6.3)	3 (15.8)	5 (9.3)	0.018
Other	2 (0.01)	0	0	15 (27.8)	na*
**Obstetric factors**					
Gestational age (days)	278 (± 14)	279 (± 11)	281 (± 8)	275 (± 22)	0.311
**Labor onset**					
Spontaneous	146 (83.4)	148 (84.1)	15 (78.9)	20 (37.0)	< 0.001
Induction	29 (16.6)	28 (15.9)	4 (21.1)	15 (27.8)	0.221
Elective cesarean section				19 (35.2)	
**Labor outcomes**					
Active secondary state (min)	32	41	41	na*	0.002
Oxytocin	107 (61.5)	123 (69.9)	13 (68.4)	29 (53.7)	0.123
Epidural	109 (62.3)	105 (59.7)	11 (57.9)	25 (46.3)	0.219
Vacuum extraction	11 (6.3)	34 (19.3)	3 (12.0)	0	0.001
Episiotomy	0	80 (45.5)	5 (26.3)	0	na*

Data are the mean ± SD or n (%).

*na = not analyzed.

Obstetrical vacuum extraction was most common in those with second-degree injuries. Mediolateral episiotomy was performed in 20.0% (85/424) of all women, resulting in iatrogenic injury in 45.5% (80/175) of the women with second-degree tears. Among women with second-degree lacerations, episiotomy was more common in deliveries by vacuum extraction (73.5% 25/34) than in spontaneous deliveries (38.7%, 55/142, p < 0.001). In the OASI group, the episiotomy rate was 26.3%, and none of the deliveries were vacuum assisted. There were no forceps deliveries.

Symptoms of prolapse were reported in 8.3% of the primiparas one year after delivery. OASI was a risk factor for developing symptoms of prolapse (aOR 7.7, Table 3). In total, 6.2% of the patients had to insert a finger in the vagina to assist in emptying their bowels.

**Table 3:** Degree of trauma and odds ratios for symptoms of prolapse, urinary incontinence, anal incontinence, sexual function and other outcomes.

Outcome measure	No injury/1st degree n = 175 (41.2%)	2nd degree n = 176 (41.6%)	OASIS n = 19 (4.5%)	Cesarean section^†^ n = 54 (12.7%)
**Adjusted OR¤ for symptoms of**				
Prolapse				
Sense of something bulging	Reference	1.4 (0.6–3.3)	7.7 (2.1–29)	0.5 (0.1–2.0)
Help empty bowel	Reference	0.7 (0.3–1.7)	1.4 (0.2–8.9)	1.0 (0.3–3.4)
Urinary incontinence				
Stress incontinence	Reference	2.6 (1.3–5.1)	2.6 (0.7–10)	0.2 (0.1–0.9)
Urge incontinence	Reference	1.2 (0.7–2.2)	4.8 (1.6–15)	0.6 (0.2–1.4)
Anal incontinence				
Any anal incontinence	Reference	0.9 (0.5–1.7)	2.8 (0.9–9.3)	0.3 (0.1–1.1)
**Crude OR for**				
Sexual function				
Pain during intercourse	Reference	1.3 (0.8–2.1)	3.1 (1.1–9.0)	0.7 (0.4–1.3)
Other				
Pain (not intercourse)	Reference	1.4 (0.7–2.7)	3.3 (1.1–10)	0.6 (0.2–1.7)
Injury affects daily life	Reference	1.2 (0.4–3.6)	18 (5.1–59)	na
Urine incontinence that affects lifestyle	Reference	1.8 (0.9–3.5)	4.6 (1.5–14)	0.3 (0.1–1.1)
Anal incontinence that affects lifestyle	Reference	2.9 (0.5–17)	23 (2.5–213)	na
Satisfied with suture of injury	Reference	0.5 (0.3–0.9)	0.3 (0.1–0.8)	na

^†^Cesarean section was compared to vaginal delivery. Data are OR (95%CI) ¤ Adjusted for age and fetal weight and BMI na = not analysed.

Urinary stress incontinence was present in 31.0% of women, and 18.0% suffered from urge incontinence. Second-degree trauma was a risk factor for stress incontinence (aOR 2.6, Table 3), and giving birth by cesarean section was protective against stress incontinence (aOR 0.2, Table 3). The risk for urge (aOR 4.7, Table 3) incontinence was elevated in the group with the largest injuries. An impact on lifestyle was reported in 12.1% of the women with urinary incontinence, and in women with OASI, the risk of urinary incontinence that affected their lifestyle was significantly elevated (Table 3).

Anal incontinence was experienced by 13.9% of women. Severe incontinence with leakage of solid stool was reported after only vaginal delivery. The severity of the symptoms was more prominent among women in the OASI group, who also had an increased risk of anal incontinence affecting their lifestyle, than among women in the other groups (OR 23, Table 3).

Most of the women were sexually active, although 9.7% of the women had not resumed sexual relations. Four women chose not to answer the question. Dyspareunia was experienced by a large proportion, accounting for 38.3% of the women who were sexually active. The rate of dyspareunia ranged from 31.3–41.4% in the groups with first/second-degree injuries and cesarean section and reached 62.5% in the group with OASI. The risk of experiencing dyspareunia was elevated in women with OASI (OR 3.1, Table 3). The feeling of being too tight (14.6%) was more common than the feeling of being too loose (8.6%).

Perineal pain was experienced by 11.6% of women, and 21 women reported that pain that was severe enough to prevent most activities in the last 3 months. This type of severe pain was present in all groups, with an elevated risk in the OASI group (OR 3.3, Table 3). OASI was a risk factor for symptoms originating from the injury that had an impact on daily activities (OR 18, Table 3). Patients with OASI had the highest rate of complications.

## Discussion

In this study, we evaluated pelvic floor function one year after birth in a nonselected cohort of primiparas and found that adverse functional outcomes were experienced in women with perineal lacerations of all grades as well as those with an intact perineum. Dyspareunia and urinary incontinence were the most common symptoms of pelvic floor dysfunction one year after childbirth. Those who suffered from OASI had a significantly higher risk of experiencing symptoms of prolapse, urinary urge incontinence, anal incontinence, dyspareunia and pain. Patients with OASI also had a significant risk of their injury impacting daily life one year after giving birth. Notably, women with moderate perineal lacerations (second-degree and episiotomy injuries) reported various symptoms of perineal dysfunction one year after delivery, and second-degree trauma was a risk factor for urinary stress incontinence.

Women who underwent cesarean section generally had a low incidence of dysfunction apart from dyspareunia (31%), for which the prevalence was in line with that in women with an intact perineum. Cesarean section was also protective against stress incontinence. The results may have been affected by the fact that this was an all-encompassing cesarean section group including women who underwent elective (35%) and emergency cesarean sections; nevertheless, the prevalence of perineal dysfunction seemed to be lower in this group than in the other groups. Similar findings have been reported in evaluations of pelvic floor function 5 and 10 years after birth, in which cesarean section deliveries were associated with significantly lower hazards for stress urinary incontinence, overactive bladder, and pelvic organ prolapse^8^. However, birth by cesarean section is associated with a risk of pelvic floor morbidity coupled to the surgery itself^9^.

Pain during intercourse was reported in 38% of the women, which is in line with data from previous studies (22.4–37.3%)^6,10^. The etiology of dyspareunia is multifactorial; scar formation after perineal laceration is one possible cause; other causes include inflammatory states, infections, and lack of sexual arousal^11^. The incidence of dyspareunia has been reported to increase after childbirth, from 12% before pregnancy to 31% six months after birth^12^. Breastfeeding has traditionally been used by midwives and gynecologists as an explanation for postpartum dyspareunia and was found to be a risk factor for dyspareunia at 6 months after birth in a recent study^13^. In Sweden, more than 70% of mothers have ceased breastfeeding at 12 months after birth^2^. To obtain in-depth knowledge of sexual function after childbirth, one needs to assess prepregnancy function and perform repeated follow-ups with a validated assessment tool, such as the pelvic organ prolapse/incontinence sexual questionnaire—IUGA revised (PISQ-IR)^14^.

In our study, the frequency of urinary incontinence was lowest in the subgroup who underwent cesarean section and was highest among patients with major injuries. Symptoms of prolapse were most prominent in patients with OASI. A previous study showed that vaginal delivery was associated with higher risks of prolapse and urinary incontinence than cesarean delivery^15^.

A similar trend was observed for anal incontinence. Excluding patients with OASI, severe anal incontinence with leakage of stool was found in only women with vaginal deliveries, which might indicate that they had undiagnosed sphincter injuries^16,17^. Various studies have postulated that the frequency of undiagnosed OASI is high, at 11–29%^16–18^. Women with OASI reported a significant impact of symptoms on daily life. Other studies have also shown that anal incontinence results in a decrease in overall quality of life^19^.

We recognize that there are limitations to our study due to the inclusion of measurements of rather subtle pelvic floor dysfunction characterized by symptoms occurring as little as once a month. There were no objective measures, such as in-person examinations, during follow-up. On the other hand, functional impairment is an easy way to identify patients who need further evaluation and treatment. In the aftermath of childbirth-related trauma, early identification of pelvic floor dysfunction enables us to intervene with effective strategies (such as pelvic floor rehabilitation) to prevent subsequent aggravation of pelvic floor dysfunction requiring surgery. Predicting and preventing long-term morbidity due to injury to the pelvic floor will also decrease healthcare costs^20^.

There are a number of biases inherent to investigations utilizing questionnaires that we tried to minimize by using a shortened version of an already existing and validated national questionnaire^21^. One possible bias is that respondents experiencing symptoms were more likely to respond, but those experiencing symptoms were comparable to the nonrespondents in the majority of baseline characteristics. The responders were older than the nonresponders, representing a potential source of responder bias given that age is a risk factor for pelvic floor dysfunction, but responder bias is unlikely due to the minor differences. Confidence intervals were wide in the OASI group due to an OASI prevalence of 4.5%, leading to a small sample size (n = 19).

The strengths of this study include its prospective cohort design and the nonselected study population since the hospital is the only one in the area. Higher fetal weight, a longer secondary active state and occiput posterior position are well-known risk factors for both OASI^22^ and levator trauma^23^. Patients who stated that they were pregnant at the time of the questionnaire were excluded from the analysis of symptoms to ensure that the results were not affected by the impact of pregnancy on function; consequently, the population eligible for analysis was substantially reduced.

The average rate of vacuum extraction in the country is 9.5%; in this study, the prevalence was 11.1%. Responders had a higher rate of vacuum delivery than nonresponders, creating possible bias due to more traumatic birth experiences. The episiotomy rate was markedly higher than the national average, at 20% in this study compared to 10% nationally^2^. On the other hand, vacuum delivery and episiotomy were not risk factors for pelvic floor dysfunction in this study. Vacuum extraction is associated with an increased prevalence of OASI and second-degree tears^24^. Episiotomy was more common in vacuum deliveries than in other types of deliveries, probably because clinical practice has been influenced by studies suggesting the reduced risk of OASI when performing episiotomy in vacuum deliveries in nulliparas^25^. The benefits and risks of routine episiotomy have been widely debated^26^. Recent studies have concluded that selective episiotomy is recommended when normal delivery is anticipated^27,28^. A reduction in the episiotomy rate in unassisted vaginal deliveries led to an increase in second-degree injuries of anatomic damage less than or equal to that caused by episiotomy without and increased rate of severe second-degree injuries^28^. There is continuing support for the use of episiotomy in vacuum delivery in nulliparas^29^ although the number needed to treat was 27, and the results from randomized controlled trials to validate these recommendations are awaited^30^. Episiotomy is an obstetric technique associated with levator injury^23^. Trauma to the levator ani during vaginal childbirth is associated with female pelvic organ prolapse^31^ and has been suggested but not confirmed to have an association with symptoms of urinary and anal incontinence^32,33^. Regarding sexual function, a recent study found no association between postpartum levator hiatus and sexual dysfunction^34^.

In conclusion, OASI is an evident risk factor for pelvic floor dysfunction after childbirth, but symptoms of pelvic floor disorder were found to be common, even in women with mild to moderate perineal laceration. Second-degree lacerations have a wide range of perineal tear extension and a subclassification may be helpful further to stratify the risk of subsequent pelvic floor dysfunction^28^. An elevated risk associated with a larger injury was observed in our study, and women with OASI reported a significant impact of their symptoms on daily life. This indicates that strategies should remain focused on preventative measures and improved diagnostics for large perineal lacerations.

Since pelvic floor disorders are stigmatizing conditions causing great physical and emotional suffering, measures aiming to reduce postpartum morbidity and to improve women's sexual health are of great importance. The association between perineal trauma and levator trauma^35^ and the increased risk of long-term pelvic dysfunction with composite trauma constitutes an important area for further research to increase confidence in vaginal births and mitigate the increased demand for cesarean sections.

## Methods

This study was part of a large interdisciplinary project involving birth-related perineal injuries, endoanal ultrasonography and postpartum pelvic floor disorders. The present study is a follow-up study in the cohort of the aforementioned project. The project aimed to prospectively include all nulliparous women who gave birth from January 2016 to January 2018 at Östersund Hospital. This is the only hospital in the county of Jämtland-Härjedalen, Sweden, and approximately 98% of pregnant women residing in the area give birth at this hospital. Written consent to participate in the study was obtained at the routine fetal anomaly scan at 18–20 weeks of pregnancy.

Women who agreed to participate in the study were clinically examined immediately after birth to diagnose perineal injury. The clinical examination was performed by a midwife or, in eligible cases, by a doctor, and consisted of inspection and bidigital palpation in the lithotomy position. The laceration grade was documented by the midwife or doctor according to the International Classification of Diseases Tenth Revision (ICD-10) World Health Organization (WHO) guidelines (Table 4). Only mediolateral episiotomy was performed. The findings were documented in a study protocol. Data on maternal, obstetric and neonatal characteristics were obtained from medical records.

**Table 4:** World Health Organization (WHO) classification of perineal tears.

Degree of tear	Perineal injury
1	Perineal skin and vaginal mucosa
2	Beyond perineal skin into muscles of perineal body
3	A. External anal sphincter (less than 50%)
	B. External anal sphincter (more than 50%)
	C. External and internal anal sphincter
4	External and internal anal sphincter; anal mucosa

Grades 3–4 are regarded as obstetric anal sphincter injury (OASI).

Participants who were one year postpartum received an invitation to complete a follow-up questionnaire by mail. An information sheet that explained the purpose of the study, security standards, data storage, and the consent process and provided the contact information of the responsible investigators was included with the letter. Participants were asked to complete a web-based questionnaire regarding symptoms of prolapse, urinary incontinence, anal incontinence, and perineal pain as well as sexual function. The questionnaire consisted of 15 questions and contained the most important items of the questionnaire for the postoperative evaluation of perineal injury provided by the National Swedish Society of Obstetrics and Gynecology^21^. The scored items of the questionnaire were validated. The questionnaire was available in only Swedish (native language). All questions had “no” or “never” as response options, and for questions about sexual function, patients had the option of not answering without being excluded from the analysis. Adaptive questioning processes were used in cases where supplementary questions were required to obtain further details on occurring symptoms, such as anal incontinence. Completion of the survey was voluntary. The patients provided informed consent by logging in with their individual codes and submitting the results. Participants received two reminders by text message and/or telephone call. Answers were automatically recorded in a database to which only the responsible investigators had access.

The participants were divided into three groups depending on the extent of perineal injury (no/first-degree injury, second-degree injury, and OASI). Women who underwent cesarean section comprised the fourth group. The period prevalence of self-reported symptoms of prolapse, urinary and fecal incontinence, dyspareunia and perineal pain were calculated based on the proportion of women who reported symptoms that matched the study definitions divided by the total number of women with available data for follow-up. In cases of multiple birth, the study considered the infant with the highest birthweight. Prolapse was defined as the feeling of something bulging out of the vagina or the need to press against the posterior vaginal wall to empty the bowel once a month or more. Stress urinary incontinence was defined as incontinence once a week to daily, urge incontinence as once a month to daily. Impact on daily activity was defined as noticeable symptoms once a month or more often. Involuntary passing of flatus was defined as the involuntary passing flatus once a month or more. Leakage of stool was defined as stool leakage once a month to daily. Sexually active was defined as intercourse within the last three months. Perineal pain was defined as pain that is impossible to ignore and that affects concentration on daily tasks and activities. The statement “very satisfied” or “satisfied” was used to determine satisfaction with suturing.

Statistical analyses were performed by analysis of variance for continuous data (ANOVA). The chi-square test or Fisher’s exact test was used as needed for categorical data, and the independent-sample t test was used for normally distributed continuous data. When analysis with the chi-square test was performed with more than two groups and statistical significance was found, pairwise testing and post hoc testing was performed to verify the finding. Odds ratios were calculated with binary logistic regression analysis, with 95% confidence intervals (CIs). Odds ratios were adjusted for age, body mass index (BMI) and fetal weight when appropriate. After ANOVA analysis, the posthoc test was performed to identify the groups that demonstrate statistically significant differences. Patients with no/first-degree injuries were compared with patients with second-degree injuries and OASI, as well as with participants who gave birth by cesarean section. Significance was set at p < 0.05. Analysis of power was performed in the initial study of the project; because the current study is a follow-up study involving the same cohort, power analysis was not applicable. All the data were compiled in an encoded database using Microsoft Access for Office 365, Version 1908 (Microsoft Corp., Redmond, WA, USA). Analyses were performed using SPSS Version 24 (IBM SPSS Statistics, Armonk, NY).

Ethical approval was granted by the Regional Ethical Review Board of Umeå (Nr. 2016–458-31 M). Informed consent was obtained from all participants. All data were deidentified prior to analysis. All experiments were performed in accordance with relevant guidelines and regulations. Patients could choose at any stage of the study to terminate their participation.

## Data Availability

The datasets generated and/or analyzed during the current study are not publicly available due to their containing information that could compromise the privacy of research participants but are available from the corresponding author upon reasonable request.
